# Endovascular bailout for closure-device failure following percutaneous endovascular aneurysm repair: A selective approach

**DOI:** 10.1016/j.jvscit.2026.102367

**Published:** 2026-06-24

**Authors:** Nivedita Mitta, Umar Sadat, Angelos Karelis, Márton Berczeli, Björn Sonesson, Nuno V. Dias

**Affiliations:** a Vascular Center, Department of Thoracic Surgery and Vascular Diseases, Skåne University Hospital, Malmö, Sweden; b Department of Clinical Sciences Malmö, Lund University, Malmö, Sweden

**Keywords:** Access-site complications, Closure-device failure, Common femoral artery, Endovascular bailout, Femoral artery dissection, Percutaneous EVAR

## Abstract

**Objective:**

Suture-mediated closure devices have enabled totally percutaneous endovascular aneurysm repair (EVAR). Although hemorrhagic access-site complications are well recognized, closure-device related common femoral artery (CFA) stenosis or dissection is less well described. We report the use of an endovascular bailout strategy for these complications and assess midterm outcomes.

**Methods:**

Between 2018 and 2023, a total of 751 consecutive percutaneous EVAR procedures were performed at a single tertiary center. Closure-device malfunction occurred in 66 cases (8.7%). Ten patients, representing 12 limbs (approximately 1.3% of all procedures), developed focal, flow-limiting CFA stenosis or dissection with controllable bleeding and were treated using an endovascular approach. Data were analyzed retrospectively from an institutional registry. Device selection was based on lesion characteristics.

**Results:**

Technical success was achieved in all cases. Bare stents were used for focal dissection or intimal flap without bleeding, whereas covered stents were used in the presence of arterial injury or contrast extravasation. At a median follow-up of 2.5 years (interquartile range, 1.6-3.5 years), imaging, mainly computed tomography angiography, showed 100% primary patency. No clinically significant restenosis or occlusion was observed. Most other closure-device failures in the cohort were managed with adjunctive hemostasis or open repair, indicating that this group represents a specific subset of complications.

**Conclusions:**

Closure-device related CFA stenosis or dissection is an uncommon but distinct complication after percutaneous EVAR. When the lesion is focal, endovascular bailout can restore flow effectively and avoid surgical exploration. This approach fits within routine EVAR practice and provides durable results at midterm follow-up.

The adoption of suture-mediated closure devices has enabled a fully percutaneous approach to endovascular aneurysm repair (EVAR).^1^ Although hemorrhagic access-site complications are well described,^2^ less attention has been directed toward the mechanical consequences of large-bore femoral access at the conclusion of EVAR, where bleeding is controlled but luminal integrity may be compromised by intimal distortion, plaque shift, or focal dissection.

Large-bore access imposes distinct mechanical demands on the common femoral artery (CFA), integrating sheath diameter, vessel depth, access angle, and suture-mediated closure forces. Hemorrhagic failure is typically evident and has traditionally mandated prompt surgical repair. Luminal compromise, in contrast, may be subtle, particularly when arterial integrity appears preserved.

In contemporary practice, established secondary access routes create an opportunity for immediate endovascular correction of such injuries without additional ipsilateral puncture, potentially avoiding surgical exposure at the end of a major aortic procedure. We describe the midterm results of a strategy prioritizing immediate endovascular correction of closure-device failure following the preclose technique.

## Methods

**Fig 1:**
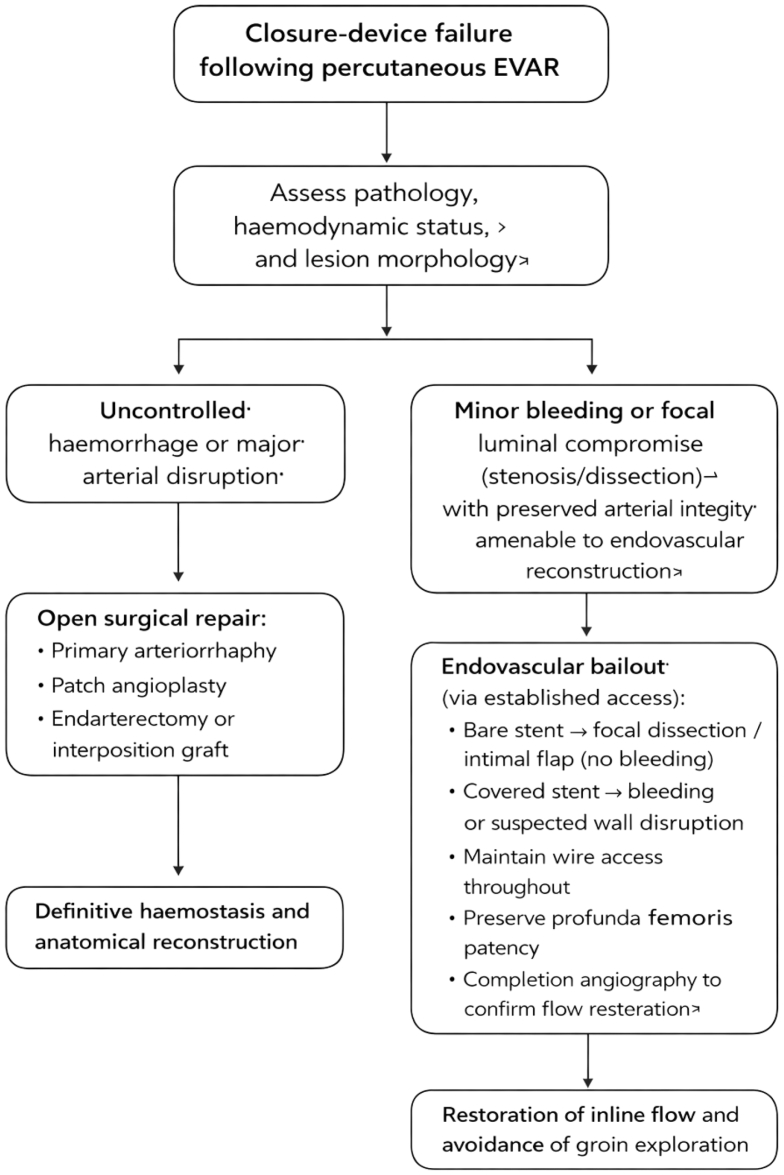
Management pathway for closure-device failure following percutaneous endovascular aneurysm repair (*EVAR*). Management algorithm for closure-device-related access complications following percutaneous EVAR. Initial assessment incorporates hemodynamic stability and lesion morphology to distinguish between uncontrolled hemorrhage or major arterial disruption, which mandates open surgical repair, and minor bleeding or focal luminal compromise with preserved arterial integrity that is amenable to endovascular bailout. Endovascular treatment is performed using established secondary access, with device selection guided by lesion characteristics: bare self-expanding stents for focal dissection or intimal flap without bleeding, and covered stent grafts where luminal compromise is associated with bleeding or suspected arterial wall disruption. Preservation of profunda femoris patency and maintenance of wire access are emphasized. Completion angiography confirms restoration of inline flow. This approach integrates into the percutaneous EVAR workflow and may avoid groin exploration in selected cases.

Management of access-site complications was not protocolized a priori but followed a consistent, pathology-driven operative logic. The resulting management pathway for closure-device failure following percutaneous EVAR is summarized in Fig 1.

Data from the institutional aortic registry (2018-2023) were reviewed to characterize the management of closure-device-related access complications across the percutaneous EVAR cohort.

Closure-device malfunction was defined as failure of percutaneous closure requiring adjunctive hemostatic, endovascular, or open intervention.

Open surgical repair or reconstruction was selected in the cases of uncontrolled hemorrhage, extensive structural compromise of the CFA, or access anatomy not amenable to safe percutaneous closure or endovascular reconstruction.

Surgical strategies included fascia suture technique or primary arteriorrhaphy where appropriate, and CFA endarterectomy with patch angioplasty or interposition grafting when required. The fascia suture technique involves suturing the cribriform fascia overlying the CFA to achieve hemostasis after large-bore femoral access.^3^

Endovascular treatment was reserved for patients in whom closure-device deployment resulted in focal, flow-limiting luminal compromise, typically stenosis or dissection, without ongoing hemorrhage or gross arterial disruption. Minor bleeding with preserved arterial integrity was incorporated into this pathway where hemostasis was controllable.

Percutaneous access was obtained under ultrasound guidance using a micropuncture technique, followed by wire exchange and upsizing to a 6F sheath. Puncture was aimed at least 1 cm above the CFA bifurcation, targeting anterior wall segments deemed suitable for percutaneous closure while avoiding heavily calcified regions where feasible.

Percutaneous access was preferred when ultrasound scan identified a safe anterior wall puncture zone suitable for suture-mediated closure. In the presence of focal CFA calcification, ultrasound imaging was used to select the least diseased anterior wall segment, avoiding calcified plaque at the intended puncture and closure-device deployment site where feasible. When no safe percutaneous window could be identified after ultrasound assessment, open femoral exposure with readiness for femoral reconstruction, including limited endarterectomy and patch repair where required, was considered.

The preclose technique was performed using two Perclose ProGlide devices (Abbott Vascular), deployed at approximately the 11 and 1 o'clock positions. A guidewire was maintained throughout large-bore sheath insertion and EVAR delivery, preserving continuous endovascular access across the CFA.

Following closure, duplex ultrasound was used to assess flow distal to the closure site, typically in the superficial femoral artery and/or profunda femoris artery. Further angiographic assessment via retained access was performed when duplex at these sites demonstrated absent or clearly abnormal distal flow, marked waveform deterioration, focal flow acceleration suggesting hemodynamically relevant stenosis, or when clinical concern persisted despite apparent hemostasis. Minor luminal irregularities without hemodynamic disturbance were not considered an indication for intervention. Cone-beam computed tomography was available in the hybrid theater and was routinely used at completion of the index EVAR procedure before closure-device deployment. It was not routinely performed after endovascular treatment of femoral access-site lesions; assessment of the bailout result was based on completion angiography. Routine EVAR follow-up imaging (computed tomography angiography and/or duplex ultrasound) was reviewed to assess stent patency.

This study was approved by the Ethics Committee for retrospective analysis (Registration No. 2014/732).

### Endovascular bailout technique

Endovascular bailout was preferentially performed using access already established during the index EVAR. In most cases, contralateral femoral up-and-over access was used; brachial access was used where already present. When one access side was anticipated to be more challenging that side was generally assessed and managed first, with wire control or alternative access maintained until satisfactory hemostasis and distal flow had been confirmed. If wire control across the injured segment had been lost, or if no established arterial access remained, access was re-established using the most appropriate available route, including contralateral crossover access, brachial access, or ipsilateral distal superficial femoral artery puncture below the lesion.

A hydrophilic 0.035-inch guidewire was advanced across the lesion and exchanged for a supportive wire. Device selection was guided by lesion morphology as follows:

- Bare-metal reconstruction was performed using short self-expanding nitinol stents, primarily Protégé EverFlex (Medtronic), for focal dissection or intimal flap without bleeding.
- Covered reconstruction was performed using self-expanding VIABAHN stent grafts (W.L. Gore & Associates, Inc) when luminal compromise was associated with contrast extravasation, persistent bleeding, or suspected arterial wall disruption.
- Completion angiography confirmed restoration of inline flow without residual stenosis or extravasation.

## Results

Between 2018 and 2023, a total of 751 consecutive percutaneous EVAR procedures were performed. Closure-device malfunction occurred in 66 cases (8.7%).

Among these, 10 patients (12 limbs; approximately 1.3% of all percutaneous EVARs) developed focal, flow-limiting CFA stenosis or dissection without uncontrolled hemorrhage, fulfilling criteria for endovascular bailout.

The majority of the remaining closure-device malfunctions were managed by adjunctive hemostasis or open surgical repair without development of flow-limiting luminal compromise requiring endovascular treatment. The present analysis, therefore, represents a selected subgroup characterized by a distinct pathological mechanism.

An established contralateral CFA or brachial approach was used in all cases. One contralateral access site required open repair, with approximately 1 L intraoperative blood loss. Two patients required bilateral bailout.

Baseline characteristics are summarized in Table. On review of preoperative imaging, the affected access vessels were generally of acceptable caliber for percutaneous access and did not show features that would have precluded a percutaneous approach. However, several demonstrated anatomical features that may reduce tolerance to closure-device deformation, including anterior wall calcification, focal plaque burden, and increased vessel depth at the puncture segment.

**Table:** Baseline anatomical and procedural characteristics of patients undergoing endovascular bailout for closure-device failure following percutaneous endovascular aneurysm repair (EVAR)

Variables	Value
No. of patients	10
No. of limbs treated	12
Age, years (median [IQR])	74 (71-78)
Female sex	9 (90)
Body mass index, kg/m² (median [IQR])	27.5 (25-34)
CFA diameter, mm (median [IQR])	8.1 (7.1-9.6)
CFA depth from skin, mm (median [IQR])	38 (28.7-45.9)
Sheath size, F (range)	18-20
Procedure type, n	EVAR (4), F/BEVAR (4), and TEVAR (2)
Stent type, limbs (n)	Bare (8), covered (3)
Bare metal stent diameter, mm (range)	7-8
Bare metal stent length, mm (range)	20-40
Covered stent diameter, mm (n)	6 (1), 7 (2)
Covered stent length, mm (n)	25 (2), 50 (1)
Bilateral bailout, patients (n)	2

*BMI,* Body mass index; *CFA,* common femoral artery; *EVAR,* endovascular aneurysm repair; *F/BEVAR,* fenestrated/branched endovascular aneurysm repair; *F,* French; *IQR*, interquartile range; *TEVAR,* thoracic endovascular aneurysm repair.

Data are presented as median [IQR] or number (%), unless otherwise specified.

The cohort was predominantly female (90%), with median body mass index 27.5 kg/m^2^ and median CFA diameter 8.1 mm. Sheath sizes ranged from 18 to 20F.

**Fig 2:**
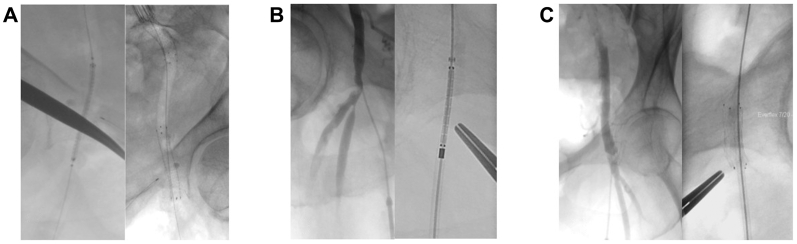
Endovascular bailout for closure device-related common femoral artery (CFA) complications following percutaneous endovascular aneurysm repair (EVAR). Representative angiographic examples demonstrating preintervention pathology and postintervention endovascular reconstruction. **A,** Focal luminal compromise following closure-device failure. The lesion demonstrates localized narrowing with associated contrast extravasation, consistent with focal arterial wall injury and intimal disruption. Carbon dioxide angiography was used to minimize iodinated-contrast exposure while confirming the site of injury. Endovascular management with deployment of a covered stent (VIABAHN; W.L. Gore & Associates, Inc) achieved effective hemostasis and restoration of luminal caliber with uninterrupted inline flow. **B,** CFA dissection with significant luminal compromise following percutaneous access. Angiography demonstrates a flow-limiting stenosis secondary to intimal flap formation. Endovascular stent placement resulted in re-expansion of the true lumen and restoration of satisfactory distal perfusion. **C,** Severe CFA luminal compromise with associated flow limitation following closure device-related arterial injury. Endovascular reconstruction using a self-expanding stent (EverFlex; Medtronic) resulted in effective luminal expansion and restoration of inline flow, avoiding the need for open surgical repair. Across all cases, endovascular bailout was performed via maintained or re-established wire access, with preservation of profunda femoris patency and confirmation of technical success on completion angiography.

Bare stents were used in eight limbs, and covered stents in three. Technical success was achieved in all cases. Representative completion angiography and follow-up imaging demonstrating stents in situ are shown in Fig 2.

At a median follow-up of 2.5 years (interquartile range, 1.6-3.5), imaging demonstrated 100% primary patency. No clinically significant restenosis or occlusion was identified. However, systematic quantification of subclinical stenosis or intimal hyperplasia was not performed.

## Discussion

This report defines a distinct access-site failure phenotype following percutaneous EVAR, characterized by focal CFA luminal compromise arising from closure-device deployment rather than primary arterial disease.

The key distinction is pathological rather than technical. Where hemorrhage predominates or arterial integrity is lost, open repair remains standard. In contrast, focal mechanical distortion focal mechanical distortion without major structural disruption defines a separate clinical entity amenable to immediate endovascular reconstruction.

Importantly, this strategy does not imply that every angiographic dissection or luminal irregularity after closure-device deployment requires treatment. In the absence of hemodynamic compromise, distal-flow disturbance, occlusion, bleeding, or convincing angiographic progression, conservative management may be appropriate. Conversely, when duplex and angiographic findings suggested flow limitation or arterial wall injury, the threshold for immediate correction was low to reduce the risk of delayed thrombosis, occlusion, or reintervention.

Endovascular bailout offers several practical advantages over open repair. It preserves skin integrity and avoids groin exploration in arteries that are frequently deep, calcified, and fragile following large-bore access. Surgical exposure in this context is technically demanding, often requires patch angioplasty, and prolongs limb ischemia time, increasing the risk of compartment syndrome. Furthermore, groin complications following prolonged procedures carry a recognized risk of infection and delayed recovery. Endovascular correction avoids these factors and allows seamless procedural completion.

The anatomical pattern observed suggests that access-site failure in this cohort may reflect a combination of vessel geometry and local wall characteristics rather than vessel diameter alone. Although CFA caliber was generally suitable for large-bore percutaneous access, several affected vessels showed anterior wall calcification, focal plaque burden, or increased depth at the puncture segment. These features may amplify the mechanical effect of suture-mediated closure by altering needle trajectory, suture capture, and focal deformation at the arteriotomy site. Given the limited number of cases, these observations should be regarded as descriptive and hypothesis-generating rather than definitive predictors.

Device selection followed a pragmatic morphology-driven approach, with bare stents used for nonhemorrhagic lesions and covered stents reserved for bleeding or arterial disruption.

Previous studies, including the Traitement des Lésions Athéromateuses de l'Artère Fémorale Commune par Technique Endovasculaire Versus Chirurgie Ouverte (TECCO) trial^4^ and subsequent analyses,^5^ demonstrate acceptable outcomes for endovascular CFA treatment. Comparable findings from European and North American series^6^^,^^7^ further support the feasibility of endovascular reconstruction in selected anatomy. However, these studies predominantly address chronic disease, whereas the present study isolates an acute, iatrogenic entity arising specifically at the conclusion of percutaneous EVAR.

The durability of femoral stenting and the feasibility of subsequent access through treated femoral segments are supported by reports such as Figueroa et al.^8^ In that series, reaccess through a previously treated CFA segment was performed using ultrasound guidance and sequential dilation, although the context was staged endoconduit use rather than closure-device injury. Their experience supports the broader principle that future femoral access may remain feasible after carefully selected endovascular reconstruction, but this should not be interpreted as direct evidence for routine repeat Perclose use through all previously stented CFAs. In our cohort, none of the patients required reintervention via the stented groins.

### Limitations

This analysis focuses on a deliberately narrow and uncommon complication, limiting statistical inference. The cohort represents a selected subgroup rather than the full spectrum of closure-device failure.

Systematic evaluation of all 66 malfunction cases, including subclinical stenosis or intimal hyperplasia, was not performed. Surveillance relied on routine EVAR imaging, and dedicated duplex follow-up was not standardized.

Management decisions were operator-dependent, and no comparative surgical cohort was included.

## Conclusions

Closure-device-induced CFA stenosis or dissection represents an uncommon but clinically relevant access complication following percutaneous EVAR. In selected cases where pathology is focal and arterial integrity preserved, endovascular bailout provides a feasible and effective solution with sustained midterm patency, while avoiding surgical re-exploration.

## Funding

None.

## Disclosures

A.K. has served on the speaker's bureau for W.L. Gore & Associates. N.V.D. has intellectual property, and serves in advisory/speaker role for Cook Medical; serves in advisory/speaker role for W.L. Gore & Associates; and serves in an advisory role for Artivion Inc.
